# Challenging the Norm: The Unrecognized Impact of Soluble Guanylyl Cyclase Subunits in Cancer

**DOI:** 10.3390/ijms251810053

**Published:** 2024-09-19

**Authors:** María Teresa L. Pino, María Victoria Rocca, Lucas H. Acosta, Jimena P. Cabilla

**Affiliations:** Centro de Altos Estudios en Ciencias Humanas y de la Salud, CONICET–Universidad Abierta Interamericana, Buenos Aires C1270AAH, Argentina; maria.pino@uai.edu.ar (M.T.L.P.); victoriarocca11@gmail.com (M.V.R.); acostalucash@gmail.com (L.H.A.)

**Keywords:** nitric oxide pathway, soluble guanylyl cyclase α1 and β1 subunits, cancer, cell signaling

## Abstract

Since the discovery of nitric oxide (NO), a long journey has led us to the present, during which much knowledge has been gained about its pathway members and their roles in physiological and various pathophysiological conditions. Soluble guanylyl cyclase (sGC), the main NO receptor composed of the sGCα1 and sGCβ1 subunits, has been one of the central figures in this narrative. However, the sGCα1 and sGCβ1 subunits remained obscured by the focus on sGC’s enzymatic activity for many years. In this review, we restore the significance of the sGCα1 and sGCβ1 subunits by compiling and analyzing available but previously overlooked information regarding their roles beyond enzymatic activity. We delve into the basics of sGC expression regulation, from its transcriptional regulation to its interaction with proteins, placing particular emphasis on evidence thus far demonstrating the actions of each sGC subunit in different tumor models. Exploring the roles of sGC subunits in cancer offers a valuable opportunity to enhance our understanding of tumor biology and discover new therapeutic avenues.

## 1. Introduction

Since nitric oxide (NO) was discovered by Murad in 1977, its many medical applications have dramatically improved people’s health around the world.

NO is involved in a wide range of physiological processes such as smooth muscle cell relaxation, neurotransmission, platelet aggregation, and host immune defense mechanisms. Also, NO and NO-derived reactive nitrogen species (RNS) participate in the generation and development of deleterious cell processes [1]. Thus, NO plays a key role in the pathology of several inflammatory diseases and other pathological conditions such as diabetes [2], neurodegenerative diseases [3,4], and cancer [5,6,7,8,9].

NO is synthesized from arginine by NO synthases (NOSs), of which three isoforms have been described: NOS1, NOS2, and NOS3.

Soluble guanylyl cyclase (sGC) is the main intracellular NO receptor and effector. The presence of sGC in most tissues was first described in the mid-1970s [10,11]. Upon binding NO, sGC synthesizes 3’,5’-cyclic guanosine monophosphate (cGMP) from GTP. Second messenger cGMP, in turn, activates several downstream targets such as cGMP-dependent protein kinases, cyclic nucleotide-gated channels, and phosphodiesterases [12,13,14].

In humans, sGC is a cytoplasmic enzyme present in almost all cells [15]. It is a heterodimeric hemoprotein composed of two subunits, α and β, of which several isoforms have been described. The role of the enzyme sGC began to be studied several years ago. Recent efforts have shifted towards understanding the role of individual sGC subunits in cancer progression. Despite notable progress, substantial gaps remain in our understanding, highlighting the complexity of this multifaceted process. In this review, we discuss the basics of sGC as a component of the NO pathway and underline the comprehensive insight into the role of sGC subunits in cancer biology.

## 2. Soluble Guanylyl Cyclase

### 2.1. Structure

**Figure 1:**
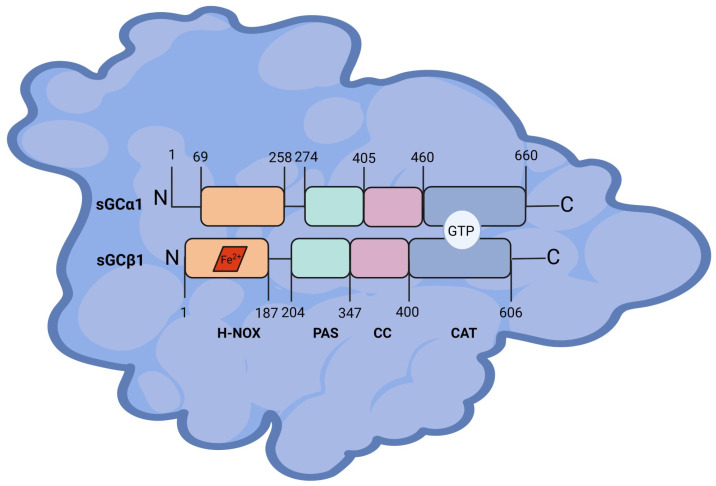
Structure of sGCα1 and sGCβ1 proteins. Heme-NO or oxygen-binding (HNOX), Per-Arnt-Sim (PAS), coiled-coil (CC), and catalytic (CAT) domains with the amino acid positions are depicted. Heme moiety is represented as a red rhombus bound to sGCβ1 subunit. Guanosine triphosphate (GTP) binding site is represented in the CAT domain. Figure created with BioRender.com.

Soluble guanylyl cyclase (sGC, EC 4.6.1.2) is an obligate heterodimer composed of two subunits, α and β. Each subunit of sGC has three functional domains: heme-binding (HNOX), dimerization, comprising Per-arnt-sim (PAS) and coiled-coil (CC) domains, and catalytic domains [16] (Figure 1). Each subunit has several isoforms; and sGCβ1 are the most widely distributed and studied and their molecular masses can be deduced from their amino acid sequences reported in the UniProt database: 77,452 Da for sGCα1 and 70,514 Da for sGCβ1 (UniProt codes Q02108 and Q02153, respectively) [17]. The sGC heterodimer α1/β1 exhibits the highest enzymatic activity [18] and is thereby considered the most physiologically relevant heterodimer.

The sGCβ2 isoform, having a molecular mass of 70,368 Da (UniProt code: O75343) [17], is more abundant in the kidney and liver. Although it can form heterodimers with sGCα1, this holoenzyme exhibits lower specific activity than its α1/β1 counterpart, although it may play a role in regulating sGC activity by competing with sGCβ1 for binding to sGCα1 [19]. Other sGCβ subunit isoforms were found in the lung [20].

The sGCα2 subunit (81,750 Da, UniProt code: P33402) forms heterodimers with sGCβ1 or sGCβ2 but exhibits less affinity for sGCβ1. The α2/β1 dimer’s specific activity corresponds to one-third of its α1/β1 counterpart [17,21]. Two additional subunits of sGC have been reported in the brain of adult humans: sGCα3 and sGCβ3. However, the existence of sGCα3 is not yet found in the NIH protein database. These share limited homology at their N-terminal ends with their respective sGCα and sGCβ isoforms but show significant homology in C-terminal regions, suggesting the existence of a common ancestor of these subunits [22]. Moreover, the preservation of the sequential arrangement of different domains within the sGC subunits, along with the similarity of sequences across these proteins in vertebrate and invertebrate animals, supports the hypothesis of a monophyletic origin of these proteins [23].

### 2.2. sGC Subunit Gene Localization and Splicing Variants

sGCα1 (GUCY1A1 or GUCY1A3) and sGCβ1 (GUCY1B1 or GUCY1B3) genes are both located in chromosome 4 sharing the same locus, whereas α2 and β2 are mapped in chromosomes 11 and 13, respectively. Splice variants of all sGCα and sGCβ subunits’ mRNA have been identified. To date, seven splicing variants have been reported for sGCα1, two for sGCα2, and six for sGCβ1 [24,25,26,27], contributing to heterogeneity in sGC subunits and conferring distinctive properties regarding activity, localization, and degradation resistance. In particular, the inhibitory sGCα2 subunit (sGCα2i) is a product of alternative RNA splicing that adds 31 amino acids to the catalytic domain, homologous to the region present in the catalytic domain of adenylate cyclases. In fact, the conversion of ATP to cAMP by sGC has been reported [28]. Although it was hypothesized that the primary function of sGCα2i was to increase the intrinsic ability of sGC to produce cAMP, subsequent evidence showed that this subunit competes with the sGCα1 and sGCα2 subunits for binding to sGCβ1 and acts as a dominant negative inhibitor. Therefore, the presence and regulation of sGCα2i expression may constitute an important modulatory mechanism of sGC activity in specific cell types [29]. sGC splicing variants may reflect tissue-specific expression and also provide a significant regulatory mechanism for its activity.

Although homodimers that may naturally form have not been isolated so far, some evidence suggests the possibility of their formation. In vitro experiments, where both subunits have been transfected, have demonstrated that the two possible homodimers are catalytically inactive and are detected in much lower proportions than heterodimers. This result may indicate the existence of a physiological equilibrium between the formation of homo- and heterodimers (which tends towards the formation of heterodimers) and could be another alternative for regulating sGC activity [30,31].

While the presence of both complementary subunits is necessary for catalytic activity, the subunits can be expressed with a different temporal pattern. In the rat brain, sGCα1 is expressed earlier than sGCβ1 during fetal brain development, suggesting that each subunit may autonomously have other functions unrelated to the classical production of cGMP [32]. Based on the work of Bidmon et al., the idea of an independent role of the sGCα2 subunit in the early stages of sensory pathway refinement began to emerge [33]. Moreover, the time-dependent expression of different sGCα1 splicing variants was shown to be crucial in embryonic stem cell differentiation [34].

### 2.3. Transcriptional Control of sGC Subunit Expression

Despite sharing the same locus, coordination of transcription of sGCα1 and sGCβ1 is still not fully understood [35]. Unlike what is observed at the level of protein sequence homology, the promoter sequences of GUCY1A1 and GUCY1B1 show limited similarity across species [36], suggesting species-specific regulatory elements and potential differences in the regulation of expression. Putative binding sites for several transcription factors, including c-myeloblastosis (c-Myb), GAGA, nuclear factor of activated T-cells (NFAT), nuclear factor kappa-light-chain-enhancer of activated B cells (NF-κB), specificity protein 1 (SP1), nuclear transcription factor Y (NFY), CCAAT-binding factors [37], and recombination signal binding protein for immunoglobulin kappa J region (RBPJ, from Notch pathway), have been found within the promoter regions of human GUCY1A1 and GUCY1B1 genes [38]. Also, other transcription factors were shown to induce the transcription of one particular subunit: SP1, cAMP-response element binding protein (CREB), and activator protein-1 (AP-1) for sGCα1 [37,39,40]. An androgen-response element (ARE) was identified upstream of exon 1 from LNCaP human prostate cancer cells and ligand-bound androgen receptor (AR) was shown to upregulate sGCα1 transcription [41].

Other experimental evidence has also shown that sGC subunit expression may be either coordinately or individually regulated.

GUCY1A1 and GUCY1B1 DNA sequences were shown to display multiple binding sites for Forkhead box subclass O (FoxO) transcription factors. It was experimentally proven that sGCα1 and sGCβ1 basal expression levels are strongly dependent on FoxO transcriptional factors since its pharmacological inhibition by AS1842856 dramatically downregulated sGCα1 and sGCβ1 mRNA expression as well as their protein levels in rat aortic smooth muscle cells [42] and renal pre-glomerular smooth muscle cells [43]. More recently, knockdown of FoxO1 and FoxO3 was shown to stimulate the transcription of both sGCα1 and sGCβ1, whereas knockdown of FoxO4 decreased their mRNA expression. FOxO-mediated effects affected both subunits’ expression to the same extent. However, these results did not concord with the protein levels, where sGCα1 was shown to be more affected than sGCβ1 [44], suggesting the participation of other post-transcriptional mechanisms, such as the interaction of mRNAs with RNA-binding proteins, such as the human antigen-R (HuR) and AUF (see below) [42].

sGCα1 and sGCβ1 expression was also shown to be specifically and directly regulated by transmembrane protease serine 2 (TMPRSS2):v-ets erythroblastosis virus E26 oncogene homolog (ERG), also known as TMPRSS2:ERG or T2E (TMPRSS2-ERG), in PCa prostate cancer cells. Two ERG-binding sites within the GUCY1A1 gene and one site within the GUCY1B1 gene have been reported in this model [45].

Also, sGCα and sGCβ subunit expression was shown to be regulated by epidermal growth factor (EGF), glucagon-like peptide 2 (GLP-2), and insulin in rat astrocytes [46]. EGF and GLP-2 decreased both sGC subunits’ expression, while insulin upregulated sGCα1 protein expression but decreased sGCβ1 levels.

Of special interest is that the sGCα1 subunit was shown to be transcriptionally upregulated by estrogen (E2) in rat pituitary gland [47,48], uterus [49], and several E2-responsive cell lines such as GH3, MCF-7, and ECC-1 [50]. The mechanism by which E2 regulates the transcription of this subunit remains unknown, since the sGCα1 gene lacks consensus sites for this hormone in its promoter region, known as estrogen-responsive elements (EREs). This characteristic adds the sGCα1 gene to 35% of the total genes regulated by E2 without ERE sequences [51,52,53]. In these cases, the mechanisms by which E2 regulates gene expression are known as indirect genomic signaling or transcriptional cross-talk, both based on the activation of gene expression mediated by the estrogen receptor (ER) without direct DNA binding [54]. Results from our laboratory have shown that sGCα1 upregulation was fully dependent on ERα, since pre-incubation with the ER inhibitor ICI 182,780 abrogates this effect in rat pituitary gland cells [47] and the GH3 lacto-somatotroph cell line [50].

It was also reported that lipopolysaccharide (LPS), interleukin-β1, and NO donors induce decreased expression of sGCα1 mRNA without altering its protein levels in pulmonary artery smooth muscle cells, presumably due to sGC’s long half-life [55].

### 2.4. Post-Transcriptional Regulation of sGC Subunits

Several studies indicate that the stability of sGCα1 and sGCβ1 mRNAs is tightly controlled in mammalian cells and varies in response to different stimuli and cell types. Elevated levels of NO, cyclic nucleotides, and growth factors have been shown to impact on sGCα1 and sGCβ1 mRNA expression [56,57,58,59,60]. Another mechanism to modulate the stability of mRNAs is the interaction of RNA-binding proteins (RBPs) to an adenine–uridine-rich element (AURE) present in the 3′ untranslated region (UTR) of the mRNA. AU-RBPs are historically categorized as functioning in mRNA post-transcriptional regulation by virtue of their ability to bind to AU-rich regions in the 3’UTR of mRNAs and mediate either mRNA degradation or stabilization [61]. Only 8% of mRNA transcripts were shown to contain these elements, suggesting that this mechanism is reserved for a variety of proteins whose expression is critical during a particular time lapse such as p53, p21, cyclin A, cyclin B1, and Chk2 (involved in DNA damage repair), among others [61]. The regulatory sequences present in the 3′ UTR regions of the mRNA of sGCα1 and sGCβ1 are critical in regulating their half-lives. The (HuR) RBP stabilizes both the mRNA of the sGCα1 subunit and the sGCβ1 subunit [48,57]. It has been demonstrated that HuR binds to target mRNAs both in the nucleus and in the cytoplasm, providing continuous protection against degradation machinery [62]. This protein binds with high affinity and specificity to the target mRNA and modifies its expression through various mechanisms, either by increasing its stability, altering its translation, or both processes simultaneously [63,64,65]. Both the mRNA of the sGCα1 subunit and the sGCβ1 subunit contain AREs in their 3’ non-coding end, and it has been demonstrated that HuR actively protects these mRNAs from degradation in rat aorta endothelial cells [57,66]. Results from our group have shown that HuR is constitutively expressed in rat pituitary gland and that E2 decreases its expression. On the other hand, the AU-rich element binding factor 1 (AUF1) protein includes among its described classical actions the destabilization of target mRNAs [67]. However, some instances were reported where it can also act by stabilizing mRNAs [68]. Several isoforms of AUF1 were shown to bind to ARE sequences in 3’UTR regions of mRNA with different affinities and to cause opposite effects on mRNA stability. Our results show that AUF1 is constitutively expressed in the rat pituitary gland and that E2 increases the levels of its mRNA. Although we have not differentiated the isoforms in this study, results obtained so far allow us to suggest that E2 increases isoforms that promote mRNA destabilization.

MicroRNAs (miRNAs) have become a fascinating field of both basic and translational biomedical research due to their impact on gene expression, widespread distribution in bodily tissues and fluids, and potential usefulness as disease biomarkers [69,70]. These 18–21-nucleotide non-coding RNAs were shown to modulate target mRNAs through recognition sites in the 3′UTR, thereby regulating their stability [71]. miRNAs regulate gene expression by two mechanisms, depending on the degree of sequence complementarity. mRNA degradation occurs after perfect complementary pairing with the target mRNA, and when base pairing is partial, mRNA translation is impeded [72].

In recent years, a few miRNAs have been reported to regulate sGC subunit expression, with most of them directly or indirectly targeting the sGCβ1 subunit. The first report on miRNA-mediated sGCβ1 regulation was published by Xu and colleagues, demonstrating a significant reduction in sGCβ1 levels in mice lungs after exposure to hypoxic conditions. Using miRNA databases, the authors selected two candidates that targeted sGCβ1: miR-34b-5p and miR-34c-5p. Both miRNAs were found to be upregulated after mice experienced hypoxia, but only miR-34c-5p significantly inhibited the luciferase activity of a reporter bearing the wildtype sGCβ1 3’UTR [73].

Park and collaborators conducted another fascinating study, demonstrating that TNF-α-induced functional impairment of vascular smooth muscle cells (VSMCs) was mediated by miR-155 upregulation, which subsequently downregulated the sGCβ1 subunit. This downregulation was observed in sera/tissue samples of patients with atherosclerosis, pre-eclampsia, and ApoE−/− mice fed a high-fat diet. The authors successfully associated sGC downregulation and VSMC dysfunction in inflammatory disease states through NF-κB-responsive miR-155, known to be implicated in endothelial dysfunction, tumor progression, atherosclerosis, and vascular inflammation and permeability. They proved that TNF-α treatment increased miR-155 expression in these cultured tissues or mouse aortic rings by activating its biogenesis through the NF-κB pathway. miR-155 was responsible for sGCβ1 downregulation, resulting in a dysfunctional sGC/cGMP axis, causing phenotypic alterations of VSMCs and impairing vascular relaxation, both of which are associated with various vascular diseases [74]. Xu and colleagues found that miR-142-5p significantly decreased sGC and cGMP levels by targeting the sGCα2 subunit in a model used to study neuropathic pain in rats with sciatic nerve injury. The significance of this study lies in the fact that the release and production of NO in the spinal cord were reported to be mechanisms related to exaggerated pain sensitivity in patients with Alzheimer’s disease. Here, the authors selected three miRNAs predicted to target sGC. Through a luciferase assay, only miR-142-5p specifically targeted the 3′-UTR of sGC mRNA, inhibiting its translation in SH-SY5Y human neuronal cells. The authors also demonstrated that miR-142-5p reduced the expression of sGC and cGMP production in this cell line. Finally, an in vivo assay showed that sGC expression, cGMP production, and neuropathic pain were significantly reduced in rats that received an orthotopic injection of adeno-associated virus carrying miR-142-5p after sciatic nerve injury [75].

Finally, Satoh and colleagues reported indirect downregulation of sGCβ1 in a rat model of metabolic syndrome, which exhibits exercise-induced pulmonary hypertension. The authors discovered that metabolic syndrome-mediated mitochondrial ROS induces miR-193b expression, which degrades the nuclear factor Y α subunit (NFYA) mRNA. NFYA is a critical transcription factor that controls the expression of the sGCβ1 subunit in pulmonary artery VSMCs. Consequently, NFYA degradation reduces sGCβ1 expression and cGMP production. Moreover, when NFYA was rescued using an adenoviral expression system in this rat model, sGCβ1 expression and cGMP levels increased, leading to a reduction in exercise-induced pulmonary hypertension [76].

In summary, sGC shows finely tuned post-transcriptional regulation, emphasizing its importance in cell physiology. Advances have been made in understanding regulation through mRNA–RBP interaction; however, miRNA-mediated regulation is still a largely unexplored field that requires thorough investigation. The evidence so far indicates that the sGCβ1 subunit is a preferential target for miRNA regulation, although it cannot be ruled out that sGCα1 may also be a target for miRNAs.

### 2.5. Post-Translational Regulation of sGC

**Figure 2:**
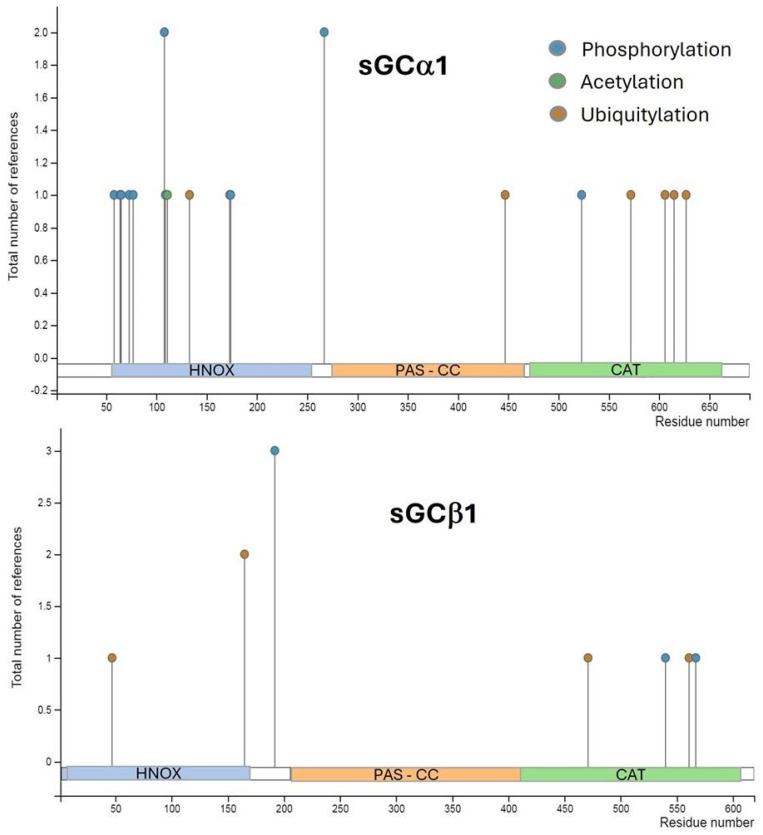
PhosphositePlus^®^ analysis of post−translational modification sites in human sGCα1 and sGCβ1 proteins as the number of records in which each modification site was assigned using proteomic discovery mass spectrometry and other methods. sGCα1, phosphorylation sites: Ser58, Thr64, Ser65, Thr73, Ser77, Ser108, Lys109, Ser173, Ser174, Ser267, and Tyr523. Acetylation site: Arg111. Ubiquitylation sites: Lys133, Lys447, Lys572, Lys606, and Lys615. sGCβ1, phosphorylation sites: Tyr192, Tyr540, and Tyr567. Ubiquitylation sites: Lys47, Lys165, Lys471, and Lys561.

Multiple putative sites for post-translational modifications, including phosphorylation, ubiquitination, and acetylation, were predicted on human sGC subunits (Figure 2). Most of them were proposed after proteomic discovery and mass spectrometry in vitro. However, the impact of these modifications on sGC activity remains unknown [77]. Little evidence on sGC phosphorylation has been reported so far in different animal models. Some authors pointed out protein kinases A and G (PKA and PKG) as candidates to phosphorylate sGC although no consensus has been reached to date and more study is required to elucidate the potential role of these kinases in modulation of sGC activity [78]. The sGCβ1 sequence was shown to contain a consensus motif for Src-like kinase phosphorylation [79]. c-Src-kinase-dependent phosphorylation of sGCβ1 at Tyr192 decreases cGMP levels by directly inhibiting sGC activity in rabbit gastric smooth muscle cells and exposes a docking site for SH2 domains, recruiting other Src-like kinases and thereby promoting multiple sGC phosphorylation [80]. Controversially, other reports indicate that Tyr192 phosphorylation is essential for recruiting other Src kinases rather than for regulation of sGC enzymatic activity [81]. Regarding sGCα1 regulation, Murthy and collaborators found no effects on sGC phosphorylation by PKA in rabbit gastric smooth muscle [80]. However, subsequent studies demonstrated that the sGCα1 subunit is a target of phosphorylation by PKA in Ser107 and Ser108 in rat pituitary cells [82]. These discrepancies could be due mainly to different experimental models and reaction times. PKG was also shown to phosphorylate the sGCα1 subunit in Ser64, thereby inhibiting sGC activity, and thus constituting a negative feedback loop [81].

It is widely recognized that cysteine (Cys) is one of the best conserved protein residues [83] whose importance in structural function has been historically highlighted [84]. However, Cys serves many other biological functions, such as catalysis, encompassing both redox-dependent and -independent processes, metal binding, and regulation of protein activities through post-translational mechanisms, which are based mainly on the reactivity of its thiol groups [85]. S-nitrosylation (SNO) is a widespread NO-driven post-translational modification that impacts over 3000 proteins [86]. This process occurs on certain Cys residues at physiological pH. Although initially considered a non-enzymatic process, compelling evidence now demonstrates that certain proteins can catalyze transnitrosylation and denitrosylation of target proteins through protein–protein interaction [87]. SNO modulates a variety of processes including protein conformations, protein–protein interactions, and other post-translational modifications [86].

In particular, sGC contains a remarkably high quantity of Cys for a cytosolic protein, many of them predicted to be accessible targets (solvent exposed) for biological and pathophysiological signaling. Experiments conducted during the 1970s and 1980s, using thiol reductants and reagents that induce disulfide bonds, established a tight relationship between cell redox state, thiols, and sGC activity [85]. sGC activity was shown to be modulated by SNO in lung cytosolic fractions from mice carrying a moderate endothelial-specific overexpression of NOS3. In this study, NO levels were shown to negatively modulate sGC activity through SNO of the sGCβ1 subunit, indicating that NO triggers a negative feedback loop regulating its own pathway [88].

Similar results were found in colonic longitudinal smooth muscle cells where proinflammatory cytokines inhibited sGC activity via iNOS-mediated SNO-sGCβ1 and induced phosphodiesterase 1A (PDE1A) expression via NF-κB with consequent cGMP hydrolysis, leading to suppression of cGMP formation and causing a decrease in muscle relaxation [89].

SNO was also reported to decrease sGC’s response to NO donors after exposure to S-nitrosoglutathione (GSNO), an S-nitrosating agent leading to a desensitization of sGC to NO in a model that used hamster cheek pouch. SNO-sGC has also been observed in human umbilical vein endothelial cells treated with vascular endothelial growth factor (VEGF) and in aorta treated with acetylcholine. When analyzing the nitrosylated Cys through mass spectrometry of purified samples of sGC treated with GSNO, the nitrosylated Cys were identified as Cys122 in the sGCβ1 subunit and Cys243 (Cys244 in humans) of the sGCα1 subunit, both located in their respective HNOX domain [90].

Another process contributing to sGC desensitization involves SNO mediated by the redox environment [87]. This form of sGC desensitization can be prevented in the presence of thioredoxin-1 (Trx1). The proposed mechanism entails the formation of a mixed disulfide bond between SNO-sGC and Cys32 of Trx1, facilitating the denitrosation of sGC and consequently releasing HNO. This mixed disulfide bond primarily targets Cys609 of the rat sGCα1 subunit, situated on the putative regulatory surface of the catalytic domain, which is constitutively S-nitrosylated. Interestingly, the authors proposed that interaction between Trx-1 and (Cys609) SNO-sGC could prevent either the SNO of Cys243 of sGCα1 and Cys122 of sGCβ1 or the conformational changes induced by SNO-sGCα1 (Cys243) and/or SNO-sGCβ1 (Cys122), ultimately leading to NO desensitization [91]. Surprisingly, the authors observed that under oxidative and nitrosative stress, the overexpression of sGC increased the levels of S-nitrosated proteins in these cell models. Although Trx1 denitrosilates sGC and prevents NO desensitization, under oxidative environment, Trx1 becomes oxidized (oTrx1) and S-nitrosated at Cys73 (SNO-oTrx1). This modification activates its ability to catalyze the transfer of its SNO to target proteins such as caspase 3 and GTPase RhoA. Under oxidative stress, SNO-sGC was reported to form a different kind of complex with oTrx1 to spread nitrosative NO signaling via this transnitrosation mechanism [92].

sGC also interacts with another member of the thioredoxin oxidoreductase superfamily named protein disulfide isomerase (PDI). This protein has the ability to isomerase mis-paired disulfides of target proteins while they are being folded in the endoplasmic reticulum. In COS-7 cell lysates, PDI inhibited NO-stimulated sGC activity whereas its redox inactive mutant did not, suggesting a sGC-redox-modulated mechanism via thiol-disulfide exchange [93].

Another protein known to interact with and modulate sGC activity is heat shock protein 70 (Hsp70). Functioning as an sGC-activating effector, this molecular chaperone synergistically activates semi-purified sGC upon co-incubation with sGC activator 3-(5′-hydroxymethyl-2′-furyl)-1-benzylindazole (YC-1) or NO. This suggests that Hsp70 interacts with sGC unlike YC-1 or NO in various in vitro and in vivo models. Interestingly, Hsp70-mediated sGC activation was shown to persist even in the presence of a NO scavenger when experiments were conducted using cytosolic fractions, thereby suggesting that this factor could activate sGC independently of NO. Additionally, co-localization of sGC and Hsp70 at the plasma membrane was reported, supporting the hypothesis that Hsp70 may facilitate sGC’s translocation to the membrane. However, despite this array of evidence, the question of whether Hsp70 is solely responsible for stimulating sGC activity remains inconclusive. This doubt arises because although the activating effect of sGC was observed in cellular fractions where both proteins co-purified, only marginal sGC activity was observed when pure Hsp70 was added. This observation suggests the possibility that the chaperone Hsp70 may require additional co-chaperones for full activation or that Hsp70 activates sGC by “rearranging” the heterodimer into a more catalytically active conformation or by increasing NO’s affinity for the heme and/or the efficacy of NO stimulation [94]. Another molecular chaperone, Hsp90, has also been described as being required for heme binding to the sGCβ1 subunit, stabilizing sGCβ1 homodimers and protecting sGC from proteasomal degradation [95]. Hsp70 and Hsp90 are well-documented members of the chaperone machinery responsible for the correct folding and trafficking of proteins related to various signal transduction pathways [96].

The study of post-translational modifications has so far focused on the context of enzymatic activity, overlooking their potential implications in other processes independent of cGMP formation.

**Figure 3:**
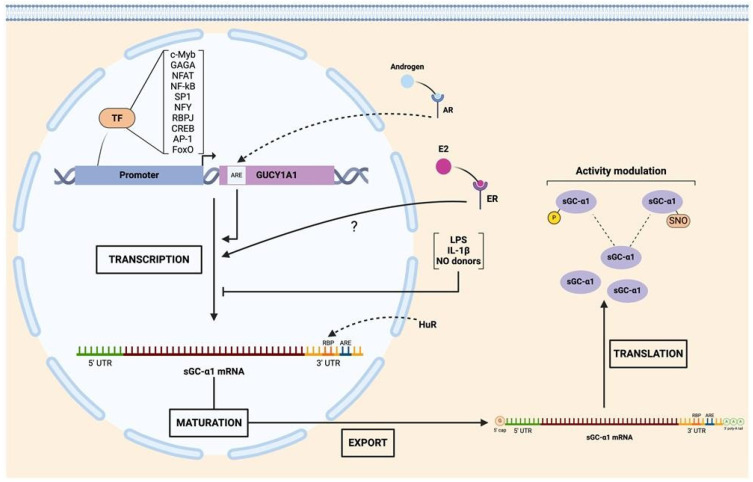
Regulation of sGCα1 expression. GUCY1A1 gene promoter is under control of several transcriptional factors (TF). GUCY1A1 gene expression can also be modulated by androgen through androgen receptor (AR) binding to androgen response elements (ARE). Estrogen (E2) modulates sGCα1 transcription by a still unknown mechanism. Inflammatory cytokines such as lipopolysaccharides (LPS) and interleukin-1β (IL-1β) as well as nitric oxide (NO) donors decrease sGCα1 mRNA expression. The human antigen-R (HuR) stabilizes sGCα1 mRNA by interacting with its 3′ untranslated region (UTR). At the protein level, sGCα1 phosphorylation (P) and S-nitrosylation (SNO) modulate sGC activity. Figure created with BioRender.com.

**Figure 4:**
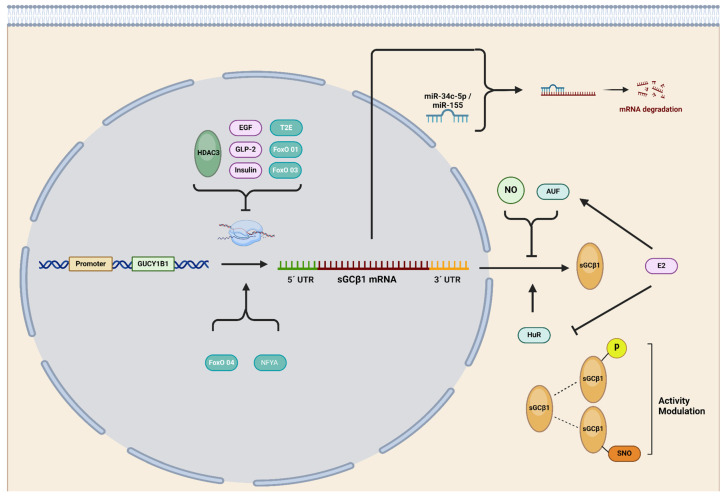
Regulation of sGCβ1 expression. GUCY1B1 gene promoter is under negative control of forkhead box proteins O1 and O3 (FoxO1 and FoxO3), TMPRSS2-ERG (T2E), and other transcription factors. GUCY1B1 gene expression can also be decreased by epidermal growth factor (EGF), glucagon-like peptide 2 (GLP2), insulin, and histone deacetylase 3 (HDAC3). FoxO4 and nuclear factor Y α subunit (NFYA) stimulate sGCβ1 expression. The human antigen-R (HuR) stabilizes sGCβ1 mRNA by interacting with its 3’ untranslated region (UTR), while AU-rich element binding factor 1 (AUF) and nitric oxide (NO) destabilize mRNA expression. Estrogen (E2) modulates sGCβ1 protein levels by modifying AU-rich element binding factor 1 (AUF1) and human antigen-R (HuR) expression. sGCβ1 mRNA is also downregulated by miR-34c-5p and miR-155. At the protein level, sGCβ1 phosphorylation (P) and S-nitrosylation (SNO) modulate sGC activity. Figure created with BioRender.com.

Graphics summarizing transcriptional, post-transcriptional, and post-translational regulation of sGCα1 and sGCβ1 are depicted in Figure 3 and Figure 4, respectively.

### 2.6. Harmony or Discord? Deciphering Interactions between sGCα1 and sGCβ1 Subunits

**Figure 5:**
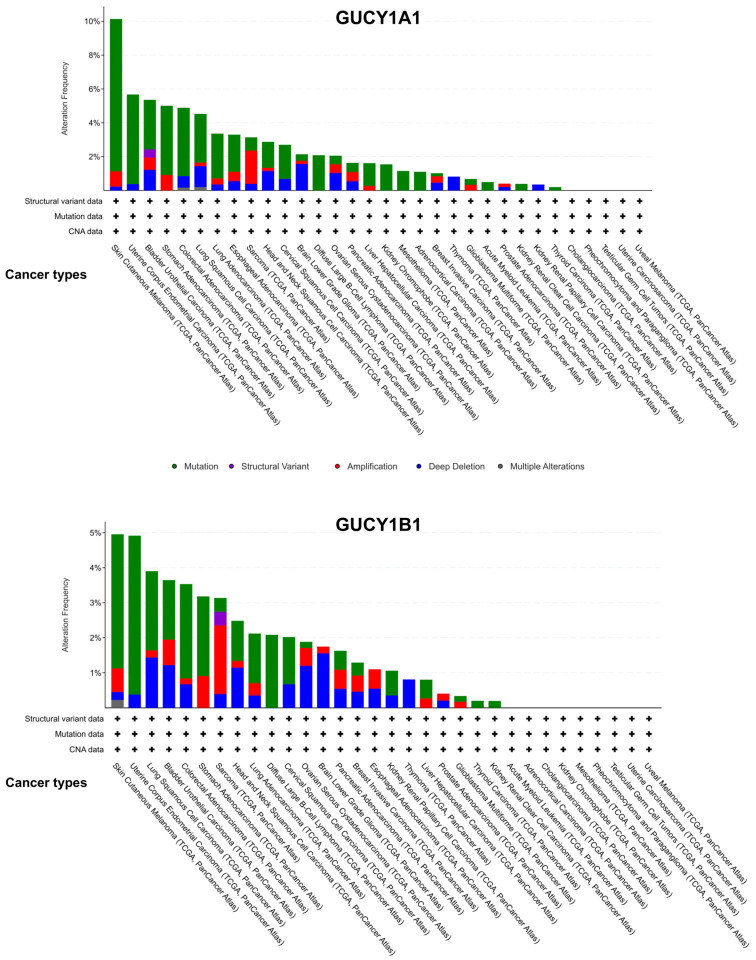
cBioPortal analysis of alteration frequency of A) GUCY1A1 and B) GUCY1B1 genes from 10,967 samples corresponding to 10,953 patients from 30 cancer types (TCGA, PanCancer Atlas). GUCY1A1 and GUCY1B1 genes are altered in 383 (3%) of queried patients. Colors represent the different gene alterations: mutation (green), structural variant (purple), amplification (red), deep deletion (blue), and multiple alterations (gray).

As discussed in this article, sGC is regulated at multiple levels: transcriptional, post-transcriptional, and post-translational. Despite its stoichiometric composition as a 1/1 heterodimeric enzyme, the α1/β1 dimer being the most active isoform, protein expression levels of sGCα1 and sGCβ1 subunits vary significantly across different tissues [15,97] and cells, even in cases where the mRNA content of both subunits was shown to be similar. This discrepancy underscores the intricate regulatory mechanisms governing sGC expression and activity. Genetically, sGCα1 and, even more so, sGCβ1 sequences (GUCY1A1 and GUCY1B1) are highly conserved across the biological scale [98], and their mutation frequency in pathological conditions such as cancer is negligible (Figure 5). However, promoter regions of GUCY1A1 and/or GUCY1B1 may differ among species [36] and are targets of diverse epigenetic modifications, such as methylation [99,100].

One important piece of evidence of sGCα1 and sGCβ1 imbalance was discovered when sGC activity was compared between metastatic and non-metastatic melanoma cells and normal melanocytes in the presence of NO donors. It was reported that NO donors could not induce cGMP production in metastatic melanoma cells, which could be due to the absence of the sGCβ1 subunit, even though sGCβ1 mRNA expression was detected and sGCα1 mRNA and protein levels were not affected [101]. The evidence supporting interregulation of sGCα and sGCβ subunits was often incidental, arising from studies with different primary aims. Perhaps for that reason, the mechanisms determining how the expression or presence/absence of one subunit impacts the expression levels of the other have not yet been fully elucidated.

To address which of all the physiological effects of the NO cascade are mediated by sGC or by cGMP in vivo, a mouse model deficient in sGC was generated by eliminating the sGCβ1 subunit (KO-sGCβ1). The results showed not only that KO-sGCβ1 mice died due to gastric obstruction and hypertension but also that sGCα1 protein levels were undetectable in lung and brain samples although its mRNA levels were similar in KO and WT animals [102]. In concordance with these findings, Mauersberger and colleagues reported that sGCα1, sGCα2, and sGCβ2 mRNA expression remained unaltered in a mouse model with a platelet-dominant KO-sGCβ1. However, protein expression of these subunits was not determined [103].

Supporting the idea of sGCα1 driving sGCβ1 expression, Bachiller et al. have demonstrated that in sGCα1-KO mice, sGCβ1 expression was reduced by 75% whereas sGCα2 expression remained unaltered. However, the authors found normal sGCβ1 expression in the aorta of these animals. These observations underline the idea of other tissue-specific factors implicated in sGC subunit regulation of expression [104]. Similar findings were provided by Zhou and colleagues. In that study, which aimed to investigate the role of TMPRSS2-ERG in NO-cGMP signaling in prostate cancer cells, silencing the sGCα1 gene expression was reported to decrease sGCβ1 protein levels without affecting its mRNA expression, and vice versa [45].

To address whether the α1/β1 and α2/β1 heterodimers could play different roles or whether one sGCα isoform could substitute the other, Mergia et al. generated knockout mice for sGCα1 (KO-sGCα1) and sGCα2 (KO-sGCα2). They found that the loss of the sGCα1 subunit but not sGCα2 was accompanied by a decrease in sGCβ1 subunit protein levels [105]. Altogether, these pieces of evidence suggest that the stable expression of each sGC subunit depends on the presence of the other. However, in U87 glioma cells, exhibiting detectable sGCβ1 levels but not sGCα1, the overexpression of sGCα1 alone produced a decrease in sGCβ1 protein amount [106]. In the same way, Postovit et al. demonstrated that in normoxia, sGCβ1 levels are above those of sGCα1, but hypoxia switched the relative abundance of sGCα1 and sGCβ1 subunits, with sGCα1 being more abundant than sGCβ1 in MDA-MB-231 breast cancer cells [107]. In the same cell line, Wen et al. found that sGCα1 and sGCβ1 protein levels were almost undetectable due to promoter methylation of both subunits. After demethylation, sGCβ1 protein levels were far higher than sGCα1 [99]. All this evidence, including the differential effects on sGC subunit expression triggered by E2 [47,48,108] and androgens [41], cannot be satisfactorily explained solely by the hypothesis of mutual stabilization of the subunits, instead suggesting the participation of other mechanisms which need to be comprehensively studied.

Another variable suggesting independent actions of both sGC subunits is their differential intracellular localization. Data from glioblastoma [109], glia [97], differentiating embryonic cells [34], and endometrial and cervical cancer cells [110] have demonstrated nuclear and cytoplasmic localization of sGCβ1, although sGCβ1 lacks a nuclear localization signal (NLS). In contrast, sGCα1 displays only cytoplasmic localization [34,97,110]. All this evidence supports the hypothesis that sGC subunits might participate in different processes as mono- or heterodimers. Furthermore, the importance of both subunit presence and compartmentalization in regulating the individual and combined actions of sGCα1 and sGCβ1 introduces several additional points of control.

## 3. sGC and cGMP in Cancer

The role of the NO pathway in tumor biology has received considerable study and is an attractive therapeutic target; indeed, several clinical trials targeting certain components of this pathway are currently underway [111]. However, the numerous pieces of evidence in the literature on the anti- or protumoral role of this pathway are controversial and paradoxical, making extrapolation and comparison difficult, not only between different tissues but also within the same experimental model. The effects of NO, both cytotoxic and cytoprotective, depend heavily on NO levels; moreover, NO-dependent components (NO/sGC/PKG/cGMP) and cGMP-independent pathways (oxidative NO pathway) vary among different tissues and cell types, which explains the variability in results. The NO pathway can promote or inhibit neoplastic transformation, tumor progression, and metastasis depending on tumor type, experimental model, and NO concentrations under experimental conditions, a phenomenon known as the “yin and yang of NO” [112,113,114]. For those interested in a deeper understanding, several excellent reviews provide comprehensive insights into this topic [111,115,116,117,118].

Most evidence seems to agree on the effects of sGC or its subunits. However, it is necessary to consider tissue and tumor heterogeneity and intrinsic variability in these systems.

Although exceptions exist, the prevailing evidence indicates that the involvement of cGMP and sGC activity in cancer biology tends towards an antitumoral effect. Several studies have demonstrated that NO donors and sGC stimulators display antitumoral actions. In breast cancer cell models, experiments with sGC and PKG activators and analogs of cGMP confirmed that sGC activity mediates growth inhibition and apoptosis of MCF-7 and MDA-MB-468 cells [119].

Similar results were obtained by Mujoo et al. They observed that the expression of NO pathway components, including sGC subunits, varies in a panel of prostate, ovary, and breast cancer cell lines. Generally, sGC activation and cGMP increase induced cell growth inhibition and apoptosis. In some cell lines, these effects were shown to be mediated by ERK1/2 phosphorylation inhibition. Notably, depending on the cell line, these deleterious effects appeared to be both dependent on and independent of cGMP [120].

Likewise, several head and neck squamous cells carcinoma cell lines were found to express critical components of the sGC/PDE/PKG signaling axis. In these models, local increases in cGMP resulting from activation of sGC or inhibition of PDE through FDA-approved drugs reduced cell viability and promoted apoptosis both in vitro and in vivo [121]. Evidence of a link between key pathways in cancer and the sGCβ1 subunit was also obtained in non-tumoral tissues. Serrano and colleagues reported that the KO of integrin-linked kinase (ILK), a kinase associated with cancer progression [122], induces upregulation of sGCβ1 in VSMCs, accompanied by an increase in the enzymatic activity of sGC. However, the potential effects on sGCα1 were not determined [123].

Loss of sGC expression in many cell lines has been proposed as an adaptive advantage in tumor progression. First, it was reported that the bradykinin receptor, a direct sGC activator, and its pathway components were expressed in androgen-dependent and -independent prostate cancer cell lines; however, sGC subunit expression was undetectable in these androgen-independent cells [124]. This observation led to the hypothesis that downregulation of sGC expression, and therefore activity, may be a canonic event in the progression towards a hormone-independent prostate tumor progression. More recently, Zhang and colleagues have demonstrated that sGC signaling is targeted in the progression from castration-sensitive to castration-resistant prostate cancer cells by two mechanisms: initially by disrupting the stoichiometry of the sGC heterodimer via sGCβ1 loss in the former, followed by recovery of sGCβ1 expression but sGC oxidative inhibition in the latter. Importantly, this shift was shown to be gradual and cell population based. In both cases, sGC activity and cGMP production were indefectibly impaired, reflecting the antitumoral role of sGC in this model [125]. Similarly, Korkmaz et al. found downregulation of both sGC subunits in tumor tissue of arterial VSMCs of oropharyngeal squamous cell carcinoma compared to adjacent, non-tumoral control [126].

Another strategy to reduce intracellular cGMP levels involves its degradation or extrusion. Upregulation of PDE5 and enhanced expression of ATP-binding cassette transporter (ABCC5)—a transporter capable of exporting anticancer drugs and cGMP out of the cytoplasm—are commonly present in many neoplasias such as pancreatic, cervical, esophageal, colorectal, breast, prostate, and leukemia [127,128,129,130,131,132].

Ovary and lung cancers seem to be the exceptions most described since cGMP was associated with cancer progression and drug resistance.

Schenk et al. postulated that sGC upregulation favored chemoresistance in some models of small cell lung cancers from patient-derived cell explants. The authors found that GUCY1A1 and GUCY1B1 were the most recurrently upregulated genes in post-chemotherapy disease progression and that increase in sGC subunit expression was dependent on Notch activation rather than amplification [133]. Analogously, in ovarian cancer, El-Sehemy and colleagues have provided evidence that Notch upregulated sGCβ1 subunit expression leading to increased cGMP production in immortalized IOSE and OVCAR ovarian cancer cells. In this sense, inhibition of sGC activity by ODQ reduced cell growth in sGC-expressing ovarian cancer cells, indicating that upregulation of sGCβ1 and increased cGMP levels might be a significant event in Notch-driven tumorigenic effects [134].

The role of sGC and cGMP in cancer has been compiled in many outstanding reviews, although most of them have been unfairly omitted due to space constraints; some of them can be found in [135,136,137,138,139,140].

## 4. Unraveling the Role of sGCα and sGCβ1 in Cancer: Insights and Implications

As discussed above, the enzyme sGC comprises the sGCα1 and sGCβ1 subunits. However, in certain cancer types, there is evidence of differing levels of these subunits, indicating a potential independent role for each in tumorigenesis and progression. Exploration of functions of sGCα1 and sGCβ1 beyond their enzymatic activity represents a novel avenue for understanding tumor biology.

### 4.1. sGCα1 in Cancer

#### 4.1.1. Prostate Cancer

Compared to other cancers, prostate cancer has received the most attention regarding the procarcinogenic effect of sGCα1. The first findings regarding sGCα1’s role in cancer were provided by the Shemshedini group who reported that androgens upregulated sGCα1 expression in prostate cancer cells. They also demonstrated that AR-driven sGCα1 upregulation was enough to drive cell proliferation, much of this effect being independent of sGCβ1, sGC activity, and NO pathway activation [41]. Noteworthily, the authors also found hyperactive AR-driven, androgen-independent sGCα1 expression that correlated with prostate cancer proliferation [141]. Interestingly, sGCα1 expression was shown to be higher in biopsies from patients with advanced prostate cancer than in those found in benign prostatic hyperplasia and normal tissue, indicating that sGCα1 could be a mediator of androgen-driven procarcinogenic effects [41]. Subsequently, the authors found that sGCα1 suppresses the transcriptional activity of specific p53-regulated genes related to apoptosis and cell survival through the formation of a protein complex constituted by sGCα1–p53 protein–protein interaction and other proteins such as COP9 signalosome subunits 4 and 5 (CSN4 and CSN5) and casein kinase 2 (CK2) in the cytoplasm. The authors suggested that this protein complex destabilizes p53 [142]. These authors also observed that the overexpression of sGCα1 confers chemoresistance. All these findings constitute a key strategy in prostate cancers that retain functional p53 [143]. Moreover, overexpression of sGCα1 was also shown to upregulate Akt protein levels and its phosphorylation state [144].

In a later report, the authors demonstrated that sGCα1 expression is positively regulated by 280B, a transcription factor overexpressed in prostate cancer that also promotes p53 degradation by upregulation of mdm2, which promotes p53 ubiquitination [144]. In order to develop a potential therapeutic application, the authors tested two peptides (A-8R and B-8R) to block sGCα1’s protumoral effects, which were able to kill both androgen-dependent and -independent prostate cancer cells expressing sGCα1 by triggering apoptosis without affecting sGCα1-non-expressing cells. Both peptides caused high ROS production. In particular, B-8R increased p53 levels and p38 activation. However, these findings failed to explain the B-8R-driven cytotoxicity [144]. Although further investigation is needed to fully understand the mechanisms involved in B-8R-mediated effects, one possible explanation is that this peptide binds an sGCα1-p53-CSN4-CSN5-CK2 complex, thereby stabilizing p53 by disrupting this protein complex [142].

#### 4.1.2. Breast Cancer

Differential expression of sGCα1 splicing variants has been reported in biopsies from malignant, benign, and normal breast tissues. Mohamadoo-Khorasani and colleagues detected three splice forms of sGCα1 and sGCβ1 in breast tissues. In malignant breast tumors, expression of sGCα1 variants lacking segments of the catalytic domain and variants of sGCβ1 lacking fragments of the HNOX domain were shown to be downregulated, although no relation was found between these findings and clinical–pathological features. Full-length mRNA transcripts and protein expression of sGCα1 were found to be higher compared to benign and normal tissues and significantly correlated with ER+/PR+/ERBB2+ tumors [145,146], tallying with the reported E2-driven upregulation of sGCα1 in other tissues [48,49].

#### 4.1.3. Endometrial and Cervical Cancer

The first evidence from our lab showed that sGC subunits were differentially and independently regulated by E2 in the rat pituitary and uterus as well as in several E2-responsive cancer cell lines [47,48,49,50,108]. The fact that E2 decreased sGC activity but increased sGCα1 expression prompted us to consider that sGCα1 could play a role in some E2-mediated effects. Also, we had reported that chronic E2—and E2-like compound—exposure caused a strong upregulation of sGCα1 that correlated with its well-known proliferative activity in the rat uterus. In view of these findings and considering that E2 plays a pivotal role in gynecological carcinogenesis [147], we hypothesized that sGCα1 could be involved in these processes.

We decided to conduct our experiments using two different cancer cell lines derived from the human uterus: ECC-1, an endometrial carcinoma cell line, and HeLa, a cervicouterine adenocarcinoma cell line. Although cervical and endometrial carcinomas exhibit characteristic features that are unique to each and differ distinctly in terms of their etiology, classification, progression, and response to therapies, they have in common that E2 plays an important role in their onset and progression [148,149,150].

We investigated the role of sGCα1 in proliferation, survival, and migration in ECC-1 (ERα+) and HeLa (ERα−) cell lines. In that work, sGCα1 knockdown significantly reduced E2-induced cell proliferation and migration and promoted cell death. These effects were observed even in the absence of E2, suggesting that sGCα1 may also mediate hormone-independent tumor growth [151]. The potential association between sGCα1 and tumor progression was previously reported by Eggen and colleagues who demonstrated that NOS2, sGCα1, and sGCα2 expression correlated with tumor growth in tissue samples from patients with early-stage cervical carcinoma [152].

#### 4.1.4. Liver Cancer

In a meta-analysis by Itkonen and colleagues, GUCY1A1 was identified as an AR- and ERG-upregulated target involved in the hexosamine biosynthesis pathway (HBP), which is overexpressed in clinical cases of prostate cancer [153]. HBP produces UDP-N-acetyl-D-glucosamine from glutamine, glucose, acetyl-Coenzyme-A, and UTP, thereby supplying substrates for the post-transcriptional modification of cytosolic and membrane proteins by O-linked β-N-acetylglucosamine transferase (OGT). Importantly, this pathway senses energy levels and links metabolic activity to the regulation of cell proliferation, with clear implications in many cancers where OGT overexpression was also reported. In a study in Bel-7402 and SMMC-7721 liver cancer cells, Yao and colleagues found that sGCα1 participated in tribbles pseudokinase 2 (TRIB2) protein-induced HBP, and O-GlcNAcylation. The authors found that TRIB2 increased sGCα1 expression by augmenting GUCY1A1 mRNA stability and impeding sGCα1 ubiquitination. Noticeably, hyperglycemia also increased sGCα1 expression. They also described the nuclear recruitment of both TRIB2 and sGCα1 after O-GlcNAcylation stimulation. In this cancer model, sGCα1 is suggested as a main component of the HBP, facilitating the activation of OGT enzyme, which catalyzes O-GlcNAcylation and activation of TRIB2, thereby contributing to the maintenance of a transformative phenotype of liver cancer cells. In turn, activated TRIB2 prevents sGCα1 ubiquitination, thereby ensuring the continuous activity of the HBP to supply substrates for O-GlcNAcylation [154]. Although the mechanisms underlying sGCα1 involvement in the HBP remains elusive, this evidence introduces a novel research avenue, indicating sGCα1’s active role in a crucial metabolic pathway in tumor biology. Moreover, it underscores sGCα1 as a key node, connecting tumorigenesis, hyperglycemia, nutrient sensing, and post-translational modification of both intracellular and secreted proteins. The resulting impact of these interactions on tumor onset and progression deserves comprehensive investigation.

**Figure 6:**
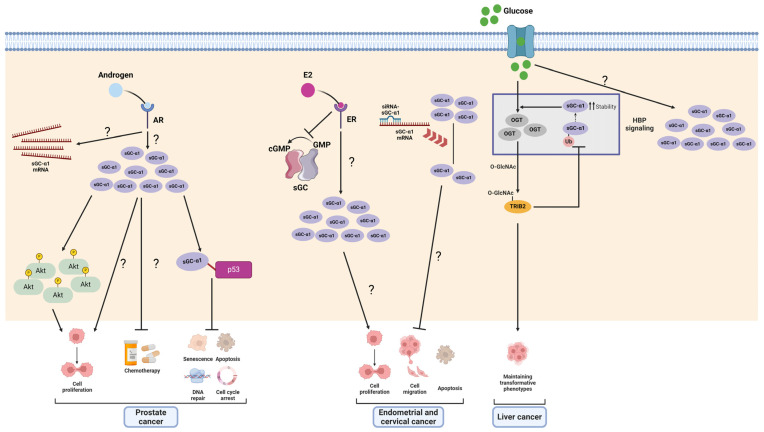
Scheme of sGCα1 actions in cancer. In prostate cancer, androgen receptor (AR) induces sGCα1 expression both at mRNA and protein levels. sGCα1 associates with p53 and suppresses its transcriptional activity, thus reducing apoptosis and other p53 downstream targets. sGCα1 also confers chemoresistance and upregulates protein kinase B (Akt) expression and activation, inducing cell proliferation. In endometrial and cervical cancer, estrogen (E2) decreases sGC activity but induces sGCα1 expression. E2 and E2-like compound-mediated sGCα1 upregulation increases cell proliferation since sGCα1 knockdown reduces cell proliferation, migration, and apoptosis. In liver cancer, high glucose levels upregulate sGCα1 expression. sGCα1 participates in the hexosamine biosynthesis pathway (HBP), providing substrates to O-linked β-N-acetylglucosamine transferase (OGT) for protein O-GlcNAcylation. OGT downstream target tribbles pseudokinase 2 (TRIB2) maintains transformative cell phenotype and stabilizes sGCα1 by inhibiting its ubiquitination. Figure created with BioRender.com.

The protumoral actions of sGCα1 are summarized in Figure 6.

### 4.2. The Emerging Role of sGCα2 as a Potential Therapeutic Target in Cancer

The role of the sGCα2 subunit in cancer remains almost completely unknown. To date, very few reports have attempted to find a link between sGCα2 expression and its role in cancer, and the evidence, though inconclusive, is controversial. First, a clinical case report on a pediatric lung adenocarcinoma with brain metastasis revealed multiple non-targetable mutations in several genes, including GUCY1A2 (coding for sGCα2), although the type and impact of those mutations were not addressed [155]. A study on biopsies from breast cancer patients demonstrated that sGCα2 protein expression is decreased in malignant tumors compared to benign and normal tissues, although its biological implications were not addressed [146]. Then, the meta-analysis and qPCR study by Li and collaborators explored the relationship between sGCα2 expression and clinically relevant parameters in gastric cancer. sGCα2 was found to be overexpressed in gastric cancer, correlating with a poor prognosis, and its overexpression was shown to correlate with histological grade and tumor stage, thus proposing sGCα2 as a potential independent prognostic marker [156]. In sum, these reports suggest that although the role of sGCα2 in cancer remains undefined, its effects could be strongly tissue-dependent.

## 5. sGCβ1 in Cancer

The understanding of sGCβ1’s role in cancer still raises numerous questions. However, ongoing investigations are suggesting that sGCβ1 may exert an antitumoral effect in various cancers, as summarized below.

### 5.1. Astrocytes, Glia, Glioma, and Neuroblastoma

The first report documenting sGCβ1’s involvement in cell proliferation was provided by Pifarre and collaborators when they found this subunit associated with chromosomes during mitosis impeding cell division in astrocyte-enriched cultures from rat cerebellum, C6 glioma cells, and a variety of cell lines. Also, when C6 cells were transiently transfected with sGCβ1 and sGCα1 or sGCα2, sGCβ1 was predominantly found in the cytoplasm, suggesting that interaction between these subunits could hinder sGCβ1 translocation to the nucleus in this model [97].

More recently, Murad and Bian’s group reported a significant decrease in sGCβ1 mRNA expression in glioma compared to normal tissue. Restoring sGCβ1 expression reduced proliferation of U6 glioma cells in vitro and in mouse xenografts. Conversely, knockdown of sGCβ1 in BE2 human neuroblastoma cells, which express normal levels of both sGC subunits, enhanced cell proliferation. Notably, these effects were independent of sGC activity, since neither inhibitors nor activators of sGC altered the proliferation rate. The authors elucidated a functional link between sGCβ1 nuclear localization and its mechanism of action in U6 cells, involving p53 accumulation, physical interaction with the TP53 gene promoter, and enhanced p53 transcriptional activity, as confirmed by increased expression of p21, a p53-responsive protein. sGCβ1 overexpression also reduced mRNA and protein levels of integrin α6, a key regulator of tumor invasion, survival, and stemness, although the mechanism underlying its downregulation remains elusive [109].

### 5.2. Breast Cancer

Wen and colleagues studied the sGCβ1 gene promoter region in two human breast cancer cells—MCF-7 and MDA-MB-231—and found they were hypermethylated. Furthermore, sGC subunit restoration reduced cell growth and induced programmed cell death in MDA-MB-231 in addition to reducing tumor incidence and tumor growth rate of MDA-MB-231 xenografts in nude mice [99]. Also, in MDA-MB-231 and in MDA-MB-468, Sotolongo and co-workers found that the sGCβ1 promoter gene region was hyperacetylated since overexpression of histone deacetylase 3 (HDAC3) significantly decreased sGCβ1 mRNA whereas the non-selective HDAC inhibitor LBH-589 had the opposite effect. Moreover, the authors performed a meta-analysis on the breast cancer database, showing that patients who exhibited higher expression of sGCβ1 had better prognosis [100]. Concordantly, Mohammadoo-Khorasani et al. reported that sGCβ1 mRNA and protein expression was lower in malignant tumors compared to benign tumors and normal breast tissues [145,146].

### 5.3. Endometrial and Cervical Cancer

We previously reported that, depending on exposition time, E2 downregulated or did not modify sGCβ1 expression in the pituitary and uterus [47,48,49,108], inversely correlating with E2-driven proliferative actions. We found that sGCβ1 expression is lower in biopsies from patients with endometrial and cervical carcinoma compared to their normal tissues, indicating that sGCβ1 could play a negative regulatory role in cancer progression. We studied the role of sGCβ1 in ECC-1 and HeLa cells, where basal expression of sGCβ1 is negligible. Overexpression of sGCβ1 reduced cell viability and increased cell death in both cell lines. sGCβ1 restoration also decreased cell migration, all these effects being independent of sGC enzymatic activity since ODQ failed to prevent sGCβ1 actions. Importantly, sGCβ1 overexpression downregulated metalloproteinase 2 (MMP-2) activity and impacted on epithelial-to-mesenchymal transition (EMT), favoring a more differentiated cell phenotype by increasing E-cadherin and decreasing N-cadherin and β-catenin protein levels. The Akt pathway is crucial for regulating many cellular processes, including cell survival, migration, and EMT. sGCβ1 restoration decreased activation of the Akt pathway by reducing phosphorylation of PDK1, Akt, PTEN, GSK-3β, and c-Raf, suggesting that sGCβ1-driven antitumoral effects could be mediated to some extent by Akt pathway downregulation. Previous evidence showed a functional interplay between the PI3k/Akt pathway and sGCβ1 in VSMCs where PDGF-mediated activation of the Akt pathway downregulated sGCβ1 expression levels [36]. The underlying mechanisms remain to be found. The nuclear localization of sGCβ1 in coincidence with previous reports also highlights the potential implication of nuclear mechanisms that should be investigated [110].

**Figure 7:**
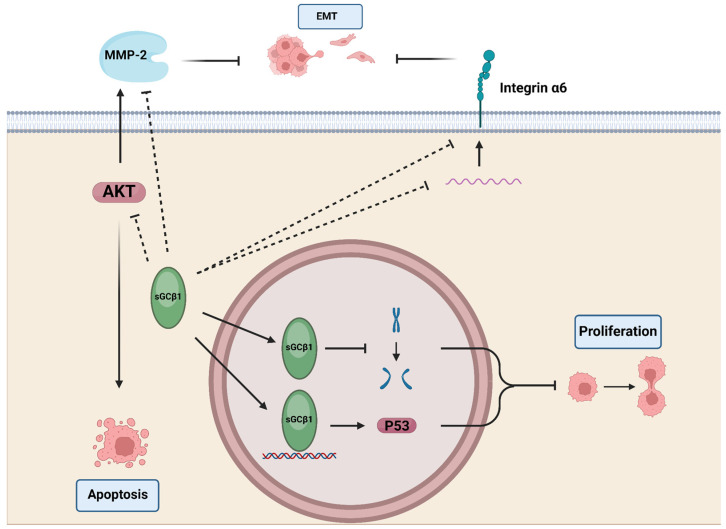
Scheme of sGCβ1 actions in cancer. Nuclear effects of sGCβ1 include physical association with chromosomes, thus impeding mitosis in glial cells. sGCβ1 increases p53 expression by interacting with the p53 promoter region in glioma cells, thus impairing cell proliferation. sGCβ1 decreases integrin α6 mRNA expression by a still unidentified mechanism in glioma cells. sGCβ1 decreases protein kinase B (Akt) pathway activation, metalloproteinase 2 (MMP-2) activity, and epithelial-to-mesenchymal transition (EMT) in endometrial and cervical cancer cells. Figure created with BioRender.com.

A graph summarizing the findings of sGCβ1 actions in cancer is provided in Figure 7.

## 6. sGCβ2 in Cancer: A Big Question Mark

As for sGCα2, evidence supporting the participation of sGCβ2 in cancer is very scarce and the connection between expression and function has not yet been elucidated.

First, it was reported that human sGCβ2 mRNA was expressed in gastric tumors but not in normal gastric tissue, suggesting that this subunit might participate in tumorigenesis [157]. A later study performed with gastric cancer samples and paired normal tissues analyzed by gene expression microarray confirmed Beherends’s observations and showed that the GUCY1B2 gene (coding for sGCβ2) was significantly upregulated in cancer samples, together with many other genes involved in tumor onset and progression, angiogenesis, migration, and microenvironment formation [158].

However, these reports suggesting a protumoral role of sGCβ2 contrast with later findings in breast cancer and in several cell lines. In breast tissue samples from patients, Mohamadoo-Khorasani and colleagues reported that sGCβ2 mRNA and protein expression was downregulated in malignant tumors compared to benign tumors and control tissues [145]. Mujoo et al. detected sGCβ2 expression in some but not all cancer cell lines tested: only PC-3 (prostate), OVCAR-3 and SK-OV-3 (ovary), and SK-Br-3 (breast) expressed sGCβ2 mRNA [120].

In either case, the biological implications of altered sGCβ2 expression remain unknown, although as seen with sGCα2, it is likely to be tissue-dependent.

## 7. Conclusions and Prospects

This review consolidates advances in understanding the roles of the sGCα1 and sGCβ1 subunits in cancer and explores potential therapeutic implications. Evidence to date demonstrates that sGC subunits, either individually or as a heterodimer, represent promising therapeutic targets. The data compiled unequivocally point out a protumoral role for sGCα1 and an antagonistic role for sGCβ1. Despite these insights, significant work remains, particularly in elucidating the specific mechanisms underlying the antagonistic actions of sGCα1 and sGCβ1 in neoplasias. Potential cross-interaction between sGCα1 and sGCβ1 subunits also remains largely unexplored and represents a critical regulatory point in many tissues.

We compiled all the current evidence comparatively from the transcriptional regulation of sGC subunits to the regulation of their half-lives. We described all the existing information on the role of each in different tumor models and their potential implications for clinical cases.

The present study curated a selection of literature from the PubMed database, acknowledging that despite our best efforts, significant works may have been omitted. Also, our search parameters encompassed publications up to April 2024; hence, subsequent contributions are not included in this review.

It would be highly advisable to avoid the technical pitfall of basing conclusions solely on the examination of a single subunit within the sGC complex. The intricate regulatory mechanisms governing its subunit levels, which include transcriptional processes and half-life, as well as differences in subcellular localization and intersubunit interactions, create numerous points of control. Neglecting the simultaneous analysis of both subunits could lead to significant misinterpretations of results.

The ubiquitous expression of sGC subunits and their regulation, both within the NO pathway and individually, unlocks multiple paths for investigation but also hinders the extrapolation of findings, underscoring the need for further research. Investigation of sGCα and sGCβ subunits as potential prognostic markers and therapeutic targets is a promising but underdeveloped area. Targeted therapies, including gene therapies specific to each subunit, may yield favorable clinical outcomes. In summary, despite numerous challenges, sGC subunits are emerging as promising therapeutic targets in cancer, warranting extensive further study.
